# The highest-cost Medicaid enrollees with sickle cell disease had annual health care expenditures nearing $200 000

**DOI:** 10.1093/haschl/qxae029

**Published:** 2024-03-11

**Authors:** Junelle Speller, Sarah Rayel, Kristen Hayashi, Michaela Kirby, Dianne Munevar, Alex Hartzman, Kevin Dietz

**Affiliations:** NORC at the University of Chicago, Chicago, IL 60603, United States; NORC at the University of Chicago, Chicago, IL 60603, United States; NORC at the University of Chicago, Chicago, IL 60603, United States; NORC at the University of Chicago, Chicago, IL 60603, United States; NORC at the University of Chicago, Chicago, IL 60603, United States; NORC at the University of Chicago, Chicago, IL 60603, United States; Dietz Analytics, Alexandria, VA 22308, United States

**Keywords:** Medicaid, Medicaid policy, gene therapy, cell and gene therapy, sickle cell disease, SCD, CGT

## Abstract

Sickle cell disease (SCD) is a painful chronic blood disorder that causes serious complications and comorbidities, often leading to premature death. SCD impacts millions of people worldwide, including an estimated 100 000 in the United States, most of whom are Black or Latino. We analyzed Medicaid enrollment, claims, and encounter data via the Transformed Medicaid Statistical Information System (T-MSIS) to examine the 2021 health care utilization and spending of Medicaid enrollees with SCD. Our analysis found that Medicaid enrollees with SCD have high annual medical and pharmacy expenditures that are not evenly distributed across the population. Among the most severe enrollees with genotypes eligible for clinical trials, those in the top 5% of health care spending incurred, on average, nearly $200 000 per year for this chronic condition.

## Introduction

Sickle cell disease (SCD) is an inherited chronic blood disorder that can cause serious complications and comorbidities, often leading to premature death. It is not uncommon for people affected by SCD to experience acute pain crises, frequent infections, bone fractures, and organ damage—complications that have far-reaching consequences for their quality of life.^1^ Sickle cell disease impacts millions of people worldwide, including an estimated 100 000 Americans, most of whom are from Black or African American and Hispanic or Latino populations.^2^

With the recent Food and Drug Administration (FDA) approval of 2 SCD gene therapy treatments, it is important to have up-to-date data on the prevalence of the disease and utilization and costs of health care services for those with SCD in the Medicaid program.^3^ Given that approximately half of the SCD population in the United States is enrolled in Medicaid, state Medicaid programs will likely play a leading role in providing access to innovative SCD disease therapies for a patient population with few options currently.^4^

### Treatment background

There are several genotypes of SCD, the most common of which are HbSS, HbSC, and HbS beta (zero and plus) thalassemia.^5^ While severity levels and complications vary by individual, the most common symptom is pain, which is the primary driver of emergency department (ED) utilization and hospital visits for people with SCD.^1^ According to the Centers for Medicare and Medicaid Services’ (CMS’) Sickle Cell Disease Action Plan, people with SCD often face other significant medical challenges, such as a higher risk of infection, stroke, and cardiac and respiratory issues, as well as stigma and other systemic obstacles that can lead to gaps in care.^4^

Prior to the recent approval of gene therapies for SCD, the only FDA-approved curative therapy for SCD was a bone marrow transplant; however, this treatment is not common due to the challenges in finding a donor match and the high risk of the procedure. Individuals with the disease most commonly rely on pain-management drugs and transfusions to treat their symptoms.^6^ The hope for a more broadly available cure was reignited in December 2023, when the FDA approved 2 potentially curative gene therapies for use by individuals 12 years and older who have the most severe forms of SCD.^3^

Despite these promising developments, stakeholders remain concerned about the high costs that Medicaid programs may incur for providing these treatments to their enrollees. Historically, gene therapies have had launch prices of around $2 million but have been indicated for rare diseases with limited eligible patient populations.^7^ While SCD is considered a rare disease, it is the most common blood disorder affecting 100 000 individuals in the United States, and the newly approved gene therapies are currently priced at $2.2 million and $3.1 million. Medicaid agencies anticipate challenges in providing essential access to these new treatments while managing budget volatility.^8^

Challenges around access to potentially curative gene therapies are particularly salient due to the specific health equity issues associated with SCD. Sickle cell disease disproportionately impacts Black Medicaid enrollees; as many as 87% of Medicaid enrollees with SCD are Black, despite making up less than 20% of Medicaid enrollment overall.^9^ Many of these individuals already face disparities in health outcomes and have also experienced gaps in access to care.^4^

To address these concerns and increase access to high-cost cell and gene therapies, the CMS Innovation Center (“Innovation Center”) announced a new Cell and Gene Therapy Access Model.^11^ The model will pool state Medicaid agencies’ bargaining power to develop outcomes-based agreements, which CMS will manage on behalf of the individual participating Medicaid programs. In February 2024, the Innovation Center held a series of webinars at which they released more details on how the model will be operationalized to increase access to cell and gene therapies in the coming years. The webinars also confirmed that the model will initially be tested with SCD gene therapies before expanding to other cell and gene therapies.^10^

To provide a comprehensive view of the population that could benefit from access to an SCD gene therapy, our analysis looks at how Medicaid enrollees with SCD currently interact with health care services and their costs.

## Data and methods

We analyzed 2021 Medicaid enrollment, claims, and encounter data available from the Transformed Medicaid Statistical Information System (T-MSIS) to examine the health care utilization and spending of Medicaid enrollees with SCD. After identifying enrollees who had been diagnosed with SCD (based on having at least 2 claims in the year with International Classification of Diseases, Tenth Revision [ICD-10], codes for SCD in any position on a claim), the enrollees were segmented into percentiles based on total health care costs in Medicaid. To more accurately estimate the costs associated with enrollees impacted by SCD, given the data limitations within T-MSIS, we excluded enrollees with missing or $0 cost data as well as enrollees with 1 or more months of comprehensive enrollment in a managed care organization in 2021. These limitations reduced the studied Medicaid SCD population from 52 524 enrollees to 12 303 unique users (23%) with reliable cost data.

Enrollees were included in this analysis if they had at least 1 claim with a positive paid amount in at least 1 category. This analysis looked at 6 different utilization categories, including inpatient hospital, ED, SCD-related prescription drugs, other prescription drugs, institutional long-term care, and all other care for patients with SCD. The SCD-related prescription drug category included voxelotor, crizanlizumab, hydroxyurea, L-glutamine, penicillin, gabapentin, pregabalin, oxycodone, hydrocodone, and morphine, as they are commonly prescribed to patients with SCD to treat pain crises. “All other care” encompasses all health care utilization captured in T-MSIS data that are not included in 1 of the other 5 categories and includes utilization of services such as outpatient non-ED services, laboratory work, and radiology, among others. Utilization and cost measures are not specific to SCD health-related utilization, with the notable exception of SCD-related prescription drugs. Utilization and costs included in this analysis are representative of the total cost of care of enrollees with SCD for Medicaid programs only.

After we calculated the total cost of care among enrollees with usable cost data and grouped those enrollees into percentile ranges by the total cost of care, we then analyzed the service-level utilization and costs of each percentile group within each diagnostic cohort. We segmented the total population of enrollees with SCD into 4 diagnostic cohorts to determine who would most likely benefit from having access to SCD gene therapies. Inclusion criteria for current clinical trials for gene therapies are based on severity and specific SCD genotypes. Accordingly, the population was first segmented by observed disease severity. Enrollees with severe SCD are defined as having 4 or more claims for crisis events in the past 24 months. Then, each severity cohort was further divided based on the genotypes included as eligibility criteria for certain clinical trials, which include HbSS, HbS beta zero thalassemia, and HbS beta plus thalassemia.

## Results

Our analysis identified 52 524 Medicaid enrollees with SCD in 2021, which aligns with prior findings that approximately 50% of the SCD population in the United States is enrolled in Medicaid.^9,4^ We found that enrollees with SCD tend to have high annual medical and pharmacy expenditures, with the average annual total cost of care for a Medicaid enrollee with SCD amounting to more than double the cost of a full year-equivalent, full-benefit Medicaid enrollee in 2021.^12^

However, these costs were not evenly distributed across the Medicaid population affected by SCD. Instead, we found that total health care costs in Medicaid were concentrated in the highest percentile ranges and among enrollees with severe SCD and clinical trial–eligible genotypes. Within this diagnostic cohort, those in the top 5% of health care spending incurred, on average, nearly $200 000 in annual costs. That same enrollee cohort had as many as 9 inpatient stays and 25 ED visits in 2021.

### Medicaid enrollees with SCD have high health care utilization; enrollees in the severe, clinical trial–eligible cohort had the highest utilization

**Figure 1.**
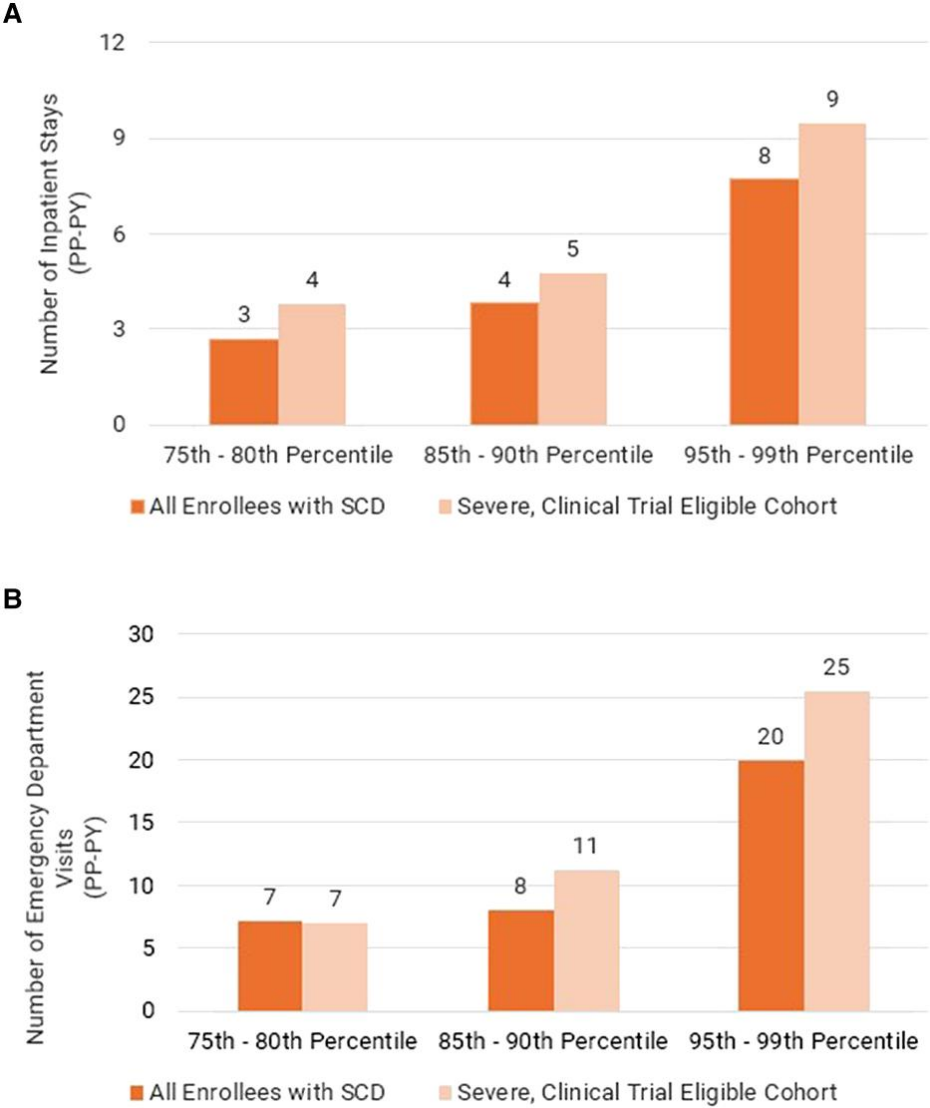
Number of inpatient stays and ED visits for Medicaid enrollees diagnosed with SCD. (A) Mean count of inpatient stays per-patient per-year of all 2021 Medicaid enrollees with a diagnosis of SCD and 2021 Medicaid enrollees with a diagnosis of SCD who are among the severe, clinical trial–eligible cohort segmented by percentiles based on total health care costs incurred in Medicaid. Average inpatient stays among cost percentiles were estimated from 2021 Medicaid enrollment, claims, and encounter data available from the Transformed Medicaid Statistical Information System (T-MSIS). (B) Mean count of ED visits per-patient per-year of all 2021 Medicaid enrollees with a diagnosis of SCD and 2021 Medicaid enrollees with diagnosis of SCD who are among the severe, clinical trial–eligible cohort segmented by percentiles based on total health care costs incurred in Medicaid. Average ED visits among cost percentile were estimated from 2021 Medicaid enrollment, claims, and encounter data available from T-MSIS. The number of inpatient stays and ED visits is an average among enrollees with at least 1 inpatient or ED claim, respectively, in the respective cohort and percentile. Abbreviations: ED, emergency department; PP-PY, per-person per-year; SCD, sickle cell disease.

The severe, clinical trial–eligible cohort had the highest utilization rates across the majority of utilization categories. This group is defined as having genotypes identified in the eligibility criteria for select clinical trials and 4 or more claims for pain crises within the past 24 months. This means that they experience the painful onset of symptoms at much higher rates than the less severe cohorts. For example, enrollees in the 95th–99th cost percentile range of the severe, clinical trial–eligible cohort had an average of 9 inpatient stays in 2021 (among those with at least 1 inpatient stay) (Figure 1A). The same cohort in the 95th–99th cost percentile range averaged 25 ED visits (among those with at least 1 ED visit) (Figure 1B). For comparison, the same cost percentile of the overall population of Medicaid enrollees with SCD had 8 inpatient stays and 20 ED visits in 2021.

### Many Medicaid enrollees with SCD have a high annual total cost of care, but the costliest enrollees are those in the severe, clinical trial–eligible cohort

**Figure 2.**
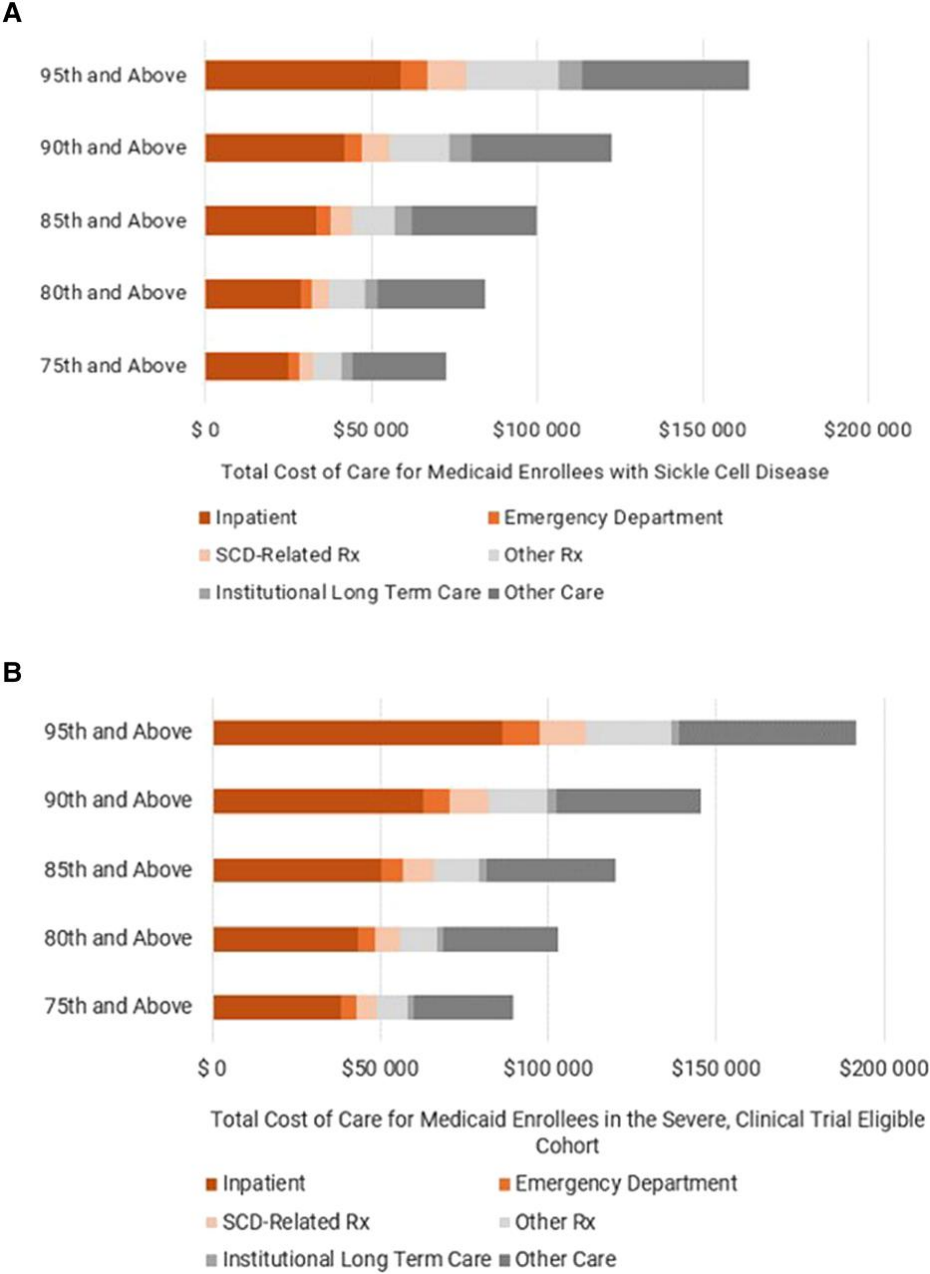
Total cost of care for Medicaid enrollees diagnosed with SCD. Total costs of care include inpatient stays, emergency department visits, prescription drugs specific to treatment of sickle cell disease, all other prescription drugs, institutional long-term care, and other care. (A) Total cost of care for all 2021 Medicaid enrollees with a diagnosis of SCD segmented by percentiles based on total health care costs incurred in Medicaid. Cost percentiles are not mutually exclusive, and each percentile range includes costs up to the 99th percentile. On average, Medicaid enrollees with SCD in the 95th–99th cost percentile incur more than $150 000 in health care costs. Average costs per percentile were calculated via an analysis of 2021 Medicaid enrollment, claims, and encounter data available from the Transformed Medicaid Statistical Information System (T-MSIS). (B) Total cost of care for 2021 Medicaid enrollees with a diagnosis of SCD who are among the severe, clinical trial–eligible cohort segmented by percentiles based on total health care costs incurred in Medicaid. Cost percentiles are not mutually exclusive, and each percentile range includes costs up to the 99th percentile. On average, Medicaid enrollees with SCD in the severe, clinical trial–eligible cohort and in the 95th–99th cost percentile incur nearly $200 000 in health care costs. Average costs per percentile were calculated via an analysis of 2021 Medicaid enrollment, claims, and encounter data available from T-MSIS. Abbreviations: Rx, prescription; SCD, sickle cell disease.

In 2021, the average total annual cost of care for a Medicaid enrollee with SCD ($22 600) was more than double the average spend for a full-year equivalent, full-benefit enrollee ($9175) in the Medicaid population overall.^12^ We also found that, while many enrollees with SCD have high health care expenditures, the costs are not evenly distributed among enrollees. Within the 95th–99th percentile range, enrollees with SCD incurred approximately $164 200 in health care costs, on average, in 2021 (Figure 2A). The high costs among the top percentiles are due to the increased utilization required to treat SCD symptoms and pain crises. There is a substantial reduction in the total cost of care between the top percentiles and those below the top quartile (data not shown).

Aside from being the largest cohort, the severe, clinical trial–eligible cohort has the highest utilization among the overall SCD population in Medicaid and, as such, is the costliest cohort. A substantial proportion of enrollees in this cohort incur Medicaid costs exceeding $100 000 in 2021 (Figure 2B). Within the severe, clinical trial–eligible cohort, enrollees in the 75th–99th percentile range undergo services that incur costs to Medicaid programs of about $89 800, on average, while those in the 95th–99th percentile undergo services that cost approximately $191 800, on average, in 2021.

Enrollees in the severe, clinical trial–eligible cohort could benefit significantly from the recently approved curative gene therapies. This group is most likely to access these therapies given their genotypes and disease severity, particularly in light of the history of Medicaid programs limiting access to new high-cost drugs to enrollees with higher severity levels.^13^

## Discussion

Sickle cell disease gene therapies have the potential to meaningfully improve the lives of Medicaid enrollees with SCD while reducing their utilization of health care services. This analysis does not attempt to compare the current costs of treating enrollees with SCD with the future costs of providing them with the new cell and gene therapies, which notably may differ from the launch prices of $2.2 million and $3.1 million based on rebates, outcomes-based contracts, and other factors. Further, curing SCD, particularly in its more severe forms, is likely to result in substantial nonfinancial benefits to enrollees, including improved quality of life and the ability to participate in more consistent employment.

Concurrently, as the Innovation Center works to develop its Cell and Gene Therapy Access Model, it is crucial to ensure that the model aims to address the systemic drivers of racial inequities as they work to increase access to therapies, especially for diseases that disproportionately impact Black individuals.^9^

## Supplementary Material

qxae029_Supplementary_Data
